# A comparative study of flat surface design and medial pivot design in posterior cruciate-retaining total knee arthroplasty: a matched pair cohort study of two years

**DOI:** 10.1186/s12891-018-2138-z

**Published:** 2018-07-18

**Authors:** Junichi Nakamura, Takaki Inoue, Toru Suguro, Masahiko Suzuki, Takahisa Sasho, Shigeo Hagiwara, Ryuichiro Akagi, Sumihisa Orita, Kazuhide Inage, Tsutomu Akazawa, Seiji Ohtori

**Affiliations:** Department of Orthopedic Surgery, Graduate School of Medicine, Chiba University, 1-8-1 Inohana, Chuo-ku, Chiba, 260-8677 Japan

**Keywords:** Total knee arthroplasty, Comparative study, Posterior cruciate-retaining, Flat surface, Medial pivot

## Abstract

**Background:**

Component design is one of the contributory factors affecting the postoperative flexion angle. The purpose of this study was to compare short-term outcomes of flat surface and medial pivot designs in posterior cruciate-retaining (CR) total knee arthroplasty (TKA).

**Methods:**

A retrospective, case-control, and observational cohort study consisted of matched-pairs of the flat surface design (Hi-Tech Knee II) and the medial pivot design (FINE Knee) in CR-TKA with a two-year follow-up period.

**Results:**

Hi-Tech Knee II and FINE knee groups each included 7 males and 38 females. Surgical time was significantly shorter in the FINE Knee group than in the Hi-Tech Knee II group (104.8 min versus 154.9 min, *p* = 0.001). Estimated total blood loss was significantly lower in the FINE Knee group than in the Hi-Tech Knee II group (654 ml versus 1158 ml, *p* = 0.001). The postoperative flexion angle was significantly better in the FINE Knee group than in the Hi-Tech Knee II group (119.3 degrees versus 112.5 degrees), and was positively correlated with the preoperative flexion angle. Postoperative Knee Society scores were significantly better in the FINE Knee group than in the Hi-Tech Knee II group (93.0 points versus 85.0 points, *p* = 0.001), especially for postoperative pain relief (46.0 points versus 39.0 points out of 50, *p* = 0.001). Complications were not observed in either group over a two-year follow-up period.

**Conclusion:**

The short-term outcome of the medial pivot design used in CR-TKA was more favorable than the flat surface design, especially for surgical time, estimated total blood loss, postoperative flexion angle, and knee pain.

**Electronic supplementary material:**

The online version of this article (10.1186/s12891-018-2138-z) contains supplementary material, which is available to authorized users.

## Background

Total knee arthroplasty (TKA) is associated with pain relief and improvement in activities of daily living. The decisions regarding whether the posterior cruciate ligament (PCL) should be replaced or retained and whether the prosthesis should be fixed using cement or not are made at the surgeon’s discretion. Studies of cruciate-retaining (CR) TKA without cement fixation of the prosthesis in patients with rheumatoid arthritis (RA) indicated prosthesis survival rates of over 90% after 10 years [1].

Hi-Tech Knee II CR-type cementless TKA (Teijin Nakashima Medical, Okayama, Japan) was developed in 1994 at Chiba University in Japan [2] and has been used for 1918 cases at 73 hospitals until 2016. This prosthesis was designed for the Japanese knee, with 6 fins at the anterior of the femoral component with the same radii in the sagittal plane, with aspect ratios (anteroposterior/mediolateral) of 0.86, PCL retention, flat-on-flat surface component geometry with 5 degrees of posterior tilt, all-polyethylene patella fixed without cement, strong initial fixation by the center screw of the tibial base plate, 10 layers of titanium alloy fiber mesh, and direct compression molded ultra-high molecular weight polyethylene (UHMWPE) [3]. The mid-term (5–12 year) results of Hi-Tech Knee II CR-type cementless TKA in 31 RA patients were satisfactory [4].

The FINE knee (Teijin Nakashima Medical, Okayama, Japan) was subsequently developed by Professor Suguro in 2001 [5]. Up to 2016, the FINE knee has been one of the most popular implants in Japan, used in 14,266 cases at 293 hospitals. The FINE knee was designed based on morphological study of Japanese normal knees to allow deeper flexion matching the Japanese lifestyle. The FINE knee has a design concept to guide tibial internal movements by medial pivot motion. The femoral component was designed to reproduce the physiologic joint line at an oblique angle of 3 degrees on the posterior condyle by osteotomy in parallel with the surgical epicondylar axis. The articular surface of the tibial medial condyle was designed to show high conformity with the femoral component and thereby enhance tibial internal rotation and secure stability during the early phase of flexion. The articular surface of the tibial lateral condyle was designed flat to allow femoral rollback, thereby allowing tibial internal rotation by medial pivot motion.

The purpose of this study was to compare short-term clinical outcomes of these two different implants with the flat surface design and the medial pivot design in CR TKA.

## Methods

The protocol for this retrospective, case-control, and observational cohort study was approved by the institutional review board. Written informed consent was obtained from all patients before surgery.

From January 2005 to December 2014, a matched-pair study was conducted for TKA. Two types of implants were compared the Hi-Tech Knee II and the FINE Knee. Inclusion criteria were a diagnosis of osteoarthritis or rheumatoid arthritis, primary TKA without previous knee surgery, CR-type component, and a minimum follow-up period of two years. Cementless fixation was applied in the Hi-Tech Knee II group and cemented fixation was used in the FINE Knee group. Osteotomy of the femur and the tibia were performed with an intramedullary guide and the proximal tibia was cut perpendicular to the axis of the tibial shaft. A posterior slope of 5 degrees was built in the tibial plates of both groups. Pairs were matched by gender and age categories (younger than 60 years, 60–64, 65–69, 70–74, 75–79, and older than 80 years). Patient condition preoperatively was documented by age, diagnosis, height, weight, body mass index (BMI, [weight]/[height]^2^), surgical approach, Knee Society score (KSS) [6], range of motion (ROM), femoro-tibial angle (FTA), joint line, and posterior condylar offset. Perioperative factors were recorded such as surgical time time and estimated total blood loss by following Gross’s formula [7]; Estimated total blood loss = estimated blood volume × (preoperative hemoglobin concentration [Hb] - postoperative day-one Hb)/(preoperative Hb + postoperative day-one Hb) × 2 + autologous blood transfusion + allogeneic blood transfusion. Estimated blood volume was calculated by 70 × body weight in men and 65 × body weight in women. Two years postoperatively, KSS, ROM, FTA, level of joint line, posterior condylar offset, α angle, β angle, γ angle, δ angle [8], and complications were compared between the Hi-Tech Knee II and FINE Knee groups.

### Statistical analysis

The Fisher’s exact probability test was calculated for differences of gender, diagnosis, and surgical approach. The Wilcoxon signed rank test was calculated for age, height, weight, BMI, KSS, ROM, and X-ray measurements with 95% confidence interval [CI]. Inter-observer and intra-observer reliability of X-ray measurements was calculated with intraclass correlation coefficients (ICC) and measurement error by minimal detectable change with Bland-Altman plot. Simple regression analysis was calculated for some correlations followed by step-wise multiple regression analysis. A *p* value less than 0.05 was considered significant (JMP Pro 12.0, SAS, North Carolina).

## Results

Hi-tech Knee II for 383 cases and FINE knee group for only 49 cases were registered. Firstly, 4 cases of FINE knee were excluded because posterior stabilizing type of knee prosthesis was applied. The remaining 45 FINE knee cases were matched to the historical cohort of Hi-tech Knee II group by gender and age categories. Patients were similar in age, BMI, and preoperative ROM in the Hi-Tech Knee II and FINE Knee groups, although height and weight were greater in the Hi-Tech Knee II group (Table 1). This study involved three consultant surgeons; A performed 53 operations, B performed 20 operations, and C performed 17 operations. All the surgeons preferred PCL retaining TKA. The mid-vastus approach was used more frequently in the FINE Knee group than in the Hi-Tech Knee II group (91% versus 64%, *p* = 0.005). The preoperative KSS indicated more severe pathology in the FINE Knee group than in the Hi-Tech Knee II group (knee score 55.1 points [95%CI, 50.9 to 59.3] versus 64.6 points [95%CI, 61.1 to 68.1], *p* = 0.005; functional score 33.3 points [95%CI, 27.2 to 39.5] versus 43.5 points [95%CI, 38.6 to 48.5], *p* = 0.013). Preoperative pain was more severe in the FINE Knee group than in the Hi-Tech Knee II group (16.9 points [95%CI, 14.1 to 19.7] versus 24.6 points [95%CI, 22.1 to 27.0] out of 50, *p* = 0.001).

**Table 1:** Patient characteristics in the Hi-Tech Knee II and FINE Knee groups

	Hi-Tech Knee II (*n* = 45)	FINE Knee (n = 45)	*p* values
Gender (male:female)	7:38	7:38	1.000^a^
Age, years	74.1 (8.1)	74.3 (10.3)	0.351^b^
Diagnosis (OA:RA)	38:7	30:15	0.846^a^
Height, cm	149.5 (8.6)	147.8(7.2)	0.015^b^
Weight, kg	60.3 (9.5)	55.7(8.4)	0.035^b^
BMI	25.8 (3.1)	25.6 (3.7)	0.566^b^
Surgical approach (Mid-vastus: Medial parapatellar)	29:16	41:4	0.005^a^
Preoperative knee score^c^	64.6 (12.0)	55.1 (14.3)	0.006^b^
Preoperative functional score^c^	45.6 (16.9)	33.3 (21.1)	0.013^b^
Preoperative total ROM, degrees	104 (23)	104 (23)	0.961^b^
Preoperative maximum flexion, degrees	114 (17)	116 (19)	0.436^b^
Preoperative flexion contracture, degrees	11 (10)	13 (10)	0.226^b^
Preoperative FTA, degrees	183.7 (6.8)	181.3 (5.2)	0.069^b^

Mean (standard deviation)

^a^Fisher’s exact probability test

^b^Wilcoxon signed rank test, ^c^Knee Society score

*OA* osteoarthritis, *RA* rheumatoid arthritis, *BMI* body mass index, *ROM* range of motion, *FTA* femoro-tibial angle

**Fig. 1:**
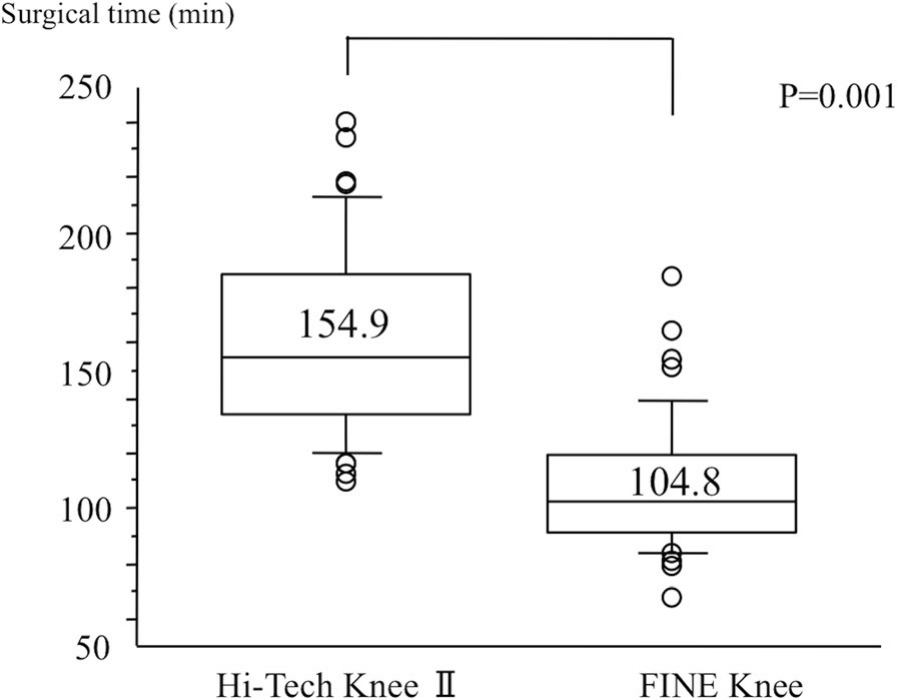
Surgical time for Hi-Tech Knee II and FINE Knee groups. Box-and-whisker plot shows the mean surgical time and standard deviation. Wilcoxon signed rank test

**Fig. 2:**
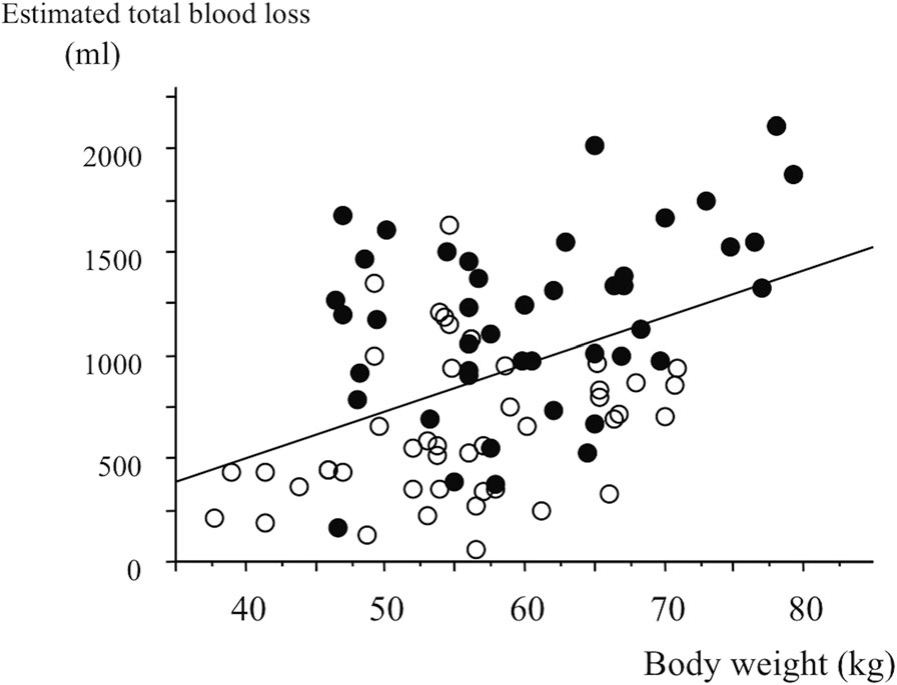
Relationship of estimated total blood loss and body weight. (Estimated total blood loss) =22 × (body weight) − 401, *R* = 0.441, *p* = 0.001, simple regression analysis. White dots: FINE Knee group and black dots: Hi-Tech Knee II group

Surgical time was significantly shorter in the FINE Knee group than in the Hi-Tech Knee II group (104.8 min [95%CI, 97.9 to 111.7] versus 154.9 min [95%CI, 145.2 to 164.6], Fig. 1). Estimated total blood loss was significantly lower in the FINE Knee group than in the Hi-Tech Knee II group (654 ml [95%CI, 550 to 758] versus 1158 ml [95%CI, 1031 to 1439], *p* = 0.001). Estimated total blood loss increased with body weight (Fig. 2).

**Fig. 3:**
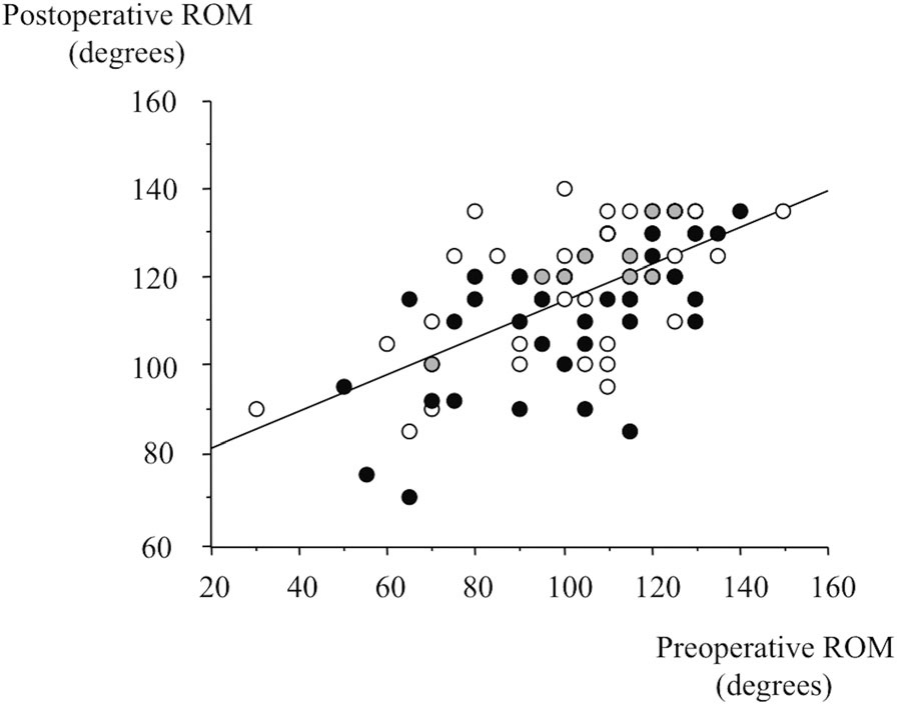
Relationship of preoperative and postoperative ROM. (Postoperative ROM) = 73 + 0.4× (preoperative ROM), *R* = 0.622, p = 0.001, simple regression analysis. White dots: FINE Knee group, black dots: Hi-Tech Knee II group, and gray dots: overlapping of FINE Knee and Hi-Tech Knee II groups

Postoperative ROM was improved in both groups, but it was significantly better in the FINE Knee group than in the Hi-Tech Knee II group (119.3 degrees [95%CI, 113.9 to 123.0] versus 112.5 degrees [95%CI, 108.1 to 117.0], *p* = 0.036). Postoperative ROM was better in patients with greater preoperative ROM (Fig. 3). FINE knee group showed a significant improvement of ROM from less than 125 degrees before to 125 degrees and more after the surgery (16 of 36 patients [44%] in FINE group versus 5 of 37 patients [14%] in Hi-tech Knee II group, *p* = 0.005, Fisher’s exact probability test, Tables 2 and 3). Postoperative knee scores of KSS were improved in both groups, but the values were significantly better in the FINE Knee group than in the Hi-Tech Knee II group (92.2 points [95%CI, 89.4 to 95.1] versus 85.0 points [95%CI, 82.7 to 87.4], *p* = 0.001). There was less postoperative pain in the FINE Knee group than in the Hi-Tech Knee II group (45.9 points [95%CI, 44.1 to 47.7] versus 39.3 points [95%CI, 37.5 to 41.2] out of 50, p = 0.001).

**Table 2:** Number of patients with 125 degrees of total ROM in Hi-tech Knee II group

		Preoperative total ROM (degrees)	
		< 125	125≤
Postoperative total ROM (degrees)	< 125	32	4
	125≤	5	4

*P* = 0.039, Fisher’s exact probability test

**Table 3:** Number of patients with 125 degrees of total ROM in Fine Knee group

		Preoperative total ROM (degrees)	
		< 125	125≤
Postoperative total ROM (degrees)	< 125	20	2
	125≤	16	7

*P* = 0.135, Fisher’s exact probability test

As for X-ray measurement, Inter-observer ICCs were 0.754 (95%CI, 0.639 to 0.835) in α angle, 0.544 (95%CI, 0.365 to 0.681) in β angle, 0.440 (95%CI, 0.258 to 0.592) in γ angle, and 0.460 (95%CI, 0.282 to 0.608) in δ angle. Intra-observer ICCs were 0.836 (95%CI, 0.760 to 0.889) in α angle, 0.827 (95%CI, 0.748 to 0.883) in β angle, 0.753 (95%CI, 0.646 to 0.831) in γ angle, and 0.602 (95%CI, 0.451 to 0.720) in δ angle. Therefore, α angle was substantial to perfect, β angle was moderate to perfect, γ angle was moderate to substantial, and δ angle was moderate to substantial. The measurement errors were 3.5 degrees in α angle, 2.9 degrees in β angle, 3.7 degrees in γ angle, and 3.9 degrees in δ angle. Postoperative X-ray findings showed that postoperative FTA was closer to normal both in the Hi-Tech Knee II and FINE Knee groups (174.8 degrees versus 174.2 degrees, *p* = 0.339). The joint line became 0.7 mm lower in the Hi-Tech Knee II group [95%CI, − 1.5 to 0.0] and 0.9 mm higher in the FINE Knee group (95%CI, 0.3 to 1.3, *p* = 0.001). Posterior condylar offset became smaller by 0.8 mm in the Hi-Tech Knee II group and 0.3 mm in the FINE Knee group (*p* = 0.802). The α angle was significantly larger in the FINE Knee group than in the Hi-Tech Knee II group (100.0 degrees [95%CI, 99.5 to 100.6] versus 95.4 degrees [95%CI, 94.9 to 95.9], p = 0.001). The β angle and γ angle were similar in the Hi-Tech Knee II and FINE Knee groups (89.1 degrees versus 88.1 degrees and 5.9 degrees versus 6.2 degrees). The δ angle was significantly smaller in the FINE Knee group than in the Hi-Tech Knee II group (87.8 degrees [95%CI, 87.0 to 88.5] versus 89.0 degrees [95%CI, 88.0 to 89.9], *p* = 0.0365).

The effect of prosthesis type and surgical approach on the duration of the surgery can be estimated by:

(Surgical time) = 168–45 × (group: 0 for Hi-Tech Knee II and 1 for FINE Knee) − 21× (approach: 0 for parapatellar and 1 for mid-vastus), R^2^ = 0.481, *p* = 0.001.

The effect of body weight and prosthesis type on total blood loss can be estimated by:

(Estimated total blood loss) =22–498 × (group: 0 for Hi-Tech Knee II and 1 for FINE Knee) + 20 × (body weight), R^2^ = 0.301, *p* = 0.001.

The effect of prosthesis type and preoperative ROM on postoperative ROM can be estimated by: (Postoperative ROM) = 69.8 + 6.5 × (group: 0 for Hi-Tech Knee II and 1 for FINE Knee) + 0.42× (preoperative ROM), R^2^ = 0.432, p = 0.001.

Complications were not observed in either group at a two-year follow-up (Additional file 1).

## Discussion

**Fig. 4:**
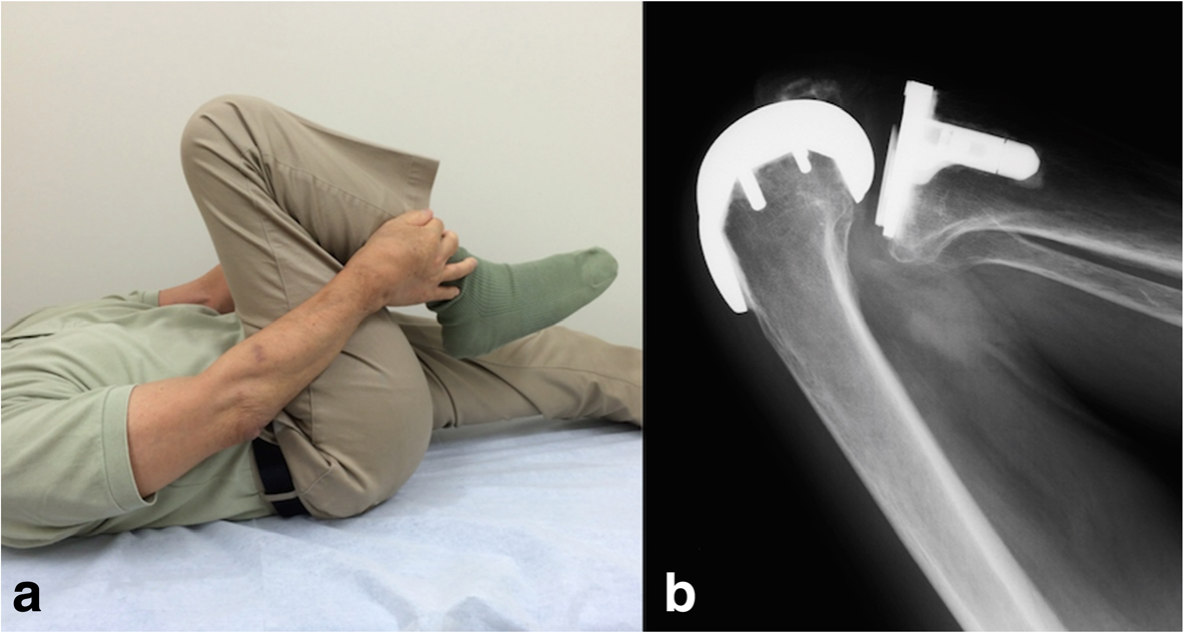
Clinical appearance (**a**) and X-ray (**b**) in a patient of FINE knee group with 140 degrees of flexion. You can see enough roll back but bone-implant impingement between the posterior femoral condyle and the posterior part of tibial plate and the polyethylene, indicating the limit of deep flexion

The two-year clinical outcome was better with the medial pivot design (FINE knee) than with the flat surface design (Hi-Tech Knee II) following CR-TKA, resulting in a 6.8 degree greater ROM. The ideal TKA would guarantee restoration of ROM. At present, 125 degrees may be enough to satisfy the patients because the much more deep flexion may lead to implant failure due to the impingement or subluxation (Fig. 4). Component design contributes to the postoperative flexion angle. Kaneyama et al. [2] performed three-dimensional kinematic analysis of the flat surface design with CR-TKA (Hi-Tech Knee II) and reported that the lateral contact point moved only slightly, but the medial contact point showed both roll-back and anterior sliding movements, with lateral pivot motions. The ten-year follow-up of the Hi-Tech Knee II shows an average flexion angle of 113.3 degrees in the medial pivot motion and 108.8 degrees in the lateral pivot motion without loosening or polyethylene wear [9]. Stiehl et al. [10] reported paradoxical movement, condylar lift-odd, and erratic screw home motion as risk factors related to abnormal wear characteristics when using a flat surface design for CR-TKA. On the other hand, rotation of the healthy knee occurs around an axis medial to the midline, and the lateral femoral condyle moves posteriorly an average of 22 mm on knee flexion to 120 degrees [11]. Magnetic resonance studies showed medial pivot motion in healthy knees and emphasized the importance of medial pivot motion in deep flexion [12]. The FINE Knee has a medially-constrained conformation to induce medial pivot motion with a postoperative flexion angle of 130.6 degrees [5]. The FINE Knee was designed to allow tibial internal rotation of 25 degrees to approximately 30 degrees, and to allow high conformity of the inside and rollback of the outside. Furthermore, the largerα angle might help the medial pivot motion and the smaller δ angle might lead the roll-back movement in the FINE knee group. We conclude that the medial pivot characteristics allow deeper flexion than the flat surface design.

Soft tissue balance plays a role in stabilizing the knee, and the choice of CR- or PS-TKA might affect the postoperative flexion angle [13]. CR-TKA can reproduce tibial rotation during flexion-extension more physiologically. The PCL kept the knee stable against distal traction force in flexion, and sacrifice of this ligament caused joint laxity [14]. Moreover, the slackness of the lateral collateral ligament makes the medial pivot motion possible [15].

The preoperative flexion angle is known to be the principal predictive factor of the postoperative angle [16]. In the current study, the postoperative ROM was better in patients with greater preoperative ROM. Other contributory factors affecting the postoperative flexion angle include both patient factors (age, comorbidity, gender, and BMI) [16, 17], and surgically modifiable factors (surgical approach, level of the joint line, tibial slope, and posterior condylar offset) [13, 17]. To equalize the patient factors, this study matched pairs by gender and age. The surgical approach did not affect postoperative flexion angle, but surgical time using the mid-vastus approach was 21 min faster than for the parapatellar approach. X-ray findings of the joint line, α angle, and δ angle were significantly different between the FINE Knee and the Hi-Tech Knee II group, although they were not predictive of the postoperative flexion angle.

The fixation method (cementless or cemented TKA) is still controversial [18]. In the Japanese registry, 83% of TKAs were cemented and 11% were cementless in 2017 [19]. Kim et al. [20] observed bone ingrowth at the surface of the titanium fiber mesh of 50.8% at 3 weeks and 62.7% at 8 weeks. Hydroxyapatite powder enhanced osteointegration to the titanium fiber [21]. Fricka et al. [22] reported equivalent survivorship at two-years in a prospective, randomized study comparing cemented versus cementless TKA. In our study, the cementless Hi-Tech Knee II group did not have any complications or revisions, but showed more estimated total blood loss than the cemented FINE knee group.

There are several limitations to this study. First, this was a short-term comparative study and long-term outcomes are unclear. However, Tamai et al. [9] reported that after 10 years, the KSS was 85.8 points in the medial pivot group and 82.9 points in the lateral pivot group when using the Hi-Tech Knee II. Yamanaka et al. [4] reported that prosthetic survival was 96.9% at 12 years postoperatively in RA patients. Long-term results of the FINE knee have not been reported. Second, because of the study’s retrospective nature, patient characteristics were heterogeneous, even though gender and age were matched. It is true that a comparative study should combine factors (disease category, surgeon, possible measurement error, pre-operative range of motion, and tilt) to match the pairs. However, it was too strict for small sample size to match the complete characteristics. Third, this study involved three consultant surgeons and permitted the surgeon’s preference. Multiple regression analysis was used to try to control confounding factors. Forth, measurement error should be taken into account. Unfortunately, the measurement error for range of motion could not be provided because of the retrospective nature in this study. As for X-ray measurement, inter-observer and intra-observer reliability was acceptable, because Brazier et al. showed radiographic measurement of the tibial psterior slope with measurement error of error of 3 degrees or more in 39.3% [23].

## Conclusion

The two-year results of the medial pivot design for CR TKA was more favorable than the flat surface design, especially for surgical time, estimated total blood loss, postoperative flexion angle, and knee pain.

## Additional file

Additional file 1: Comprehensive data table of patient characteristics in the Hi-Tech Knee II and FINE Knee groups. (XLSX 31 kb)

## Abbreviations

BMI: body mass index
CR: cruciate-retaining
FTA: femoro-tibial angle
Hb: hemoglobin concentration
KSS: Knee Society score
PCL: posterior cruciate ligament
RA: rheumatoid arthritis
ROM: range of motion
TKA: total knee arthroplasty
